# Therapeutic Efficacy of Nanodrug Delivery Compared With Current Nonsurgical Treatments for Patients With Cutaneous Melanoma: A Systematic Review

**DOI:** 10.7759/cureus.107513

**Published:** 2026-04-21

**Authors:** Zarish Hussain, Nujood Buhaimed, Maryam Al Khalifa, Jawaher Aljalahma, Bedoor Alomran

**Affiliations:** 1 Medicine, King Hamad University Hospital, Royal Medical Services, Busaiteen, BHR; 2 Medicine, Bahrain Defence Force Hospital, Royal Medical Services, Riffa, BHR; 3 Dermatology, Bahrain Defence Force Hospital, Royal Medical Services, Riffa, BHR; 4 Radiology, Bahrain Defence Force Hospital, Royal Medical Services, Riffa, BHR

**Keywords:** nano drug delivery system, nanoparticle, stage iii melanoma, stage iv melanoma, vaccine

## Abstract

Melanoma is an aggressive skin cancer characterized by high metastatic potential and drug resistance with limited responses to available therapies. Therefore, further research regarding treatments and technologies, such as nanoparticles for cutaneous melanoma, is essential.

The objective of this systematic review was to summarize published evidence on the safety and efficacy of using nanoparticles with cutaneous melanoma treatments to reduce tumor recurrence and/or survival outcomes for melanoma patients.

A literature search of PubMed, Scopus, Cochrane Library, ClinicalTrials.gov, and Google Scholar was conducted from January 2008 to January 2023. Publications describing randomized and nonrandomized clinical trials of nanoparticle cutaneous melanoma treatment were included. The risk of bias for the included studies was rated according to the Cochrane Handbook, and the strength of the clinical data and subsequent melanoma treatment recommendations were graded according to the Oxford Center for Evidence-Based Medicine Levels of Evidence.

Seven studies were included, which involved 825 adult patients. Most of these patients had stage III/IV cutaneous melanoma. The outcomes measured across the studies were immune response, progression-free survival, tumor response rate, and toxicity. Both the MelQbg10 vaccine and nab-paclitaxel induced positive responses in patients.

Novel nanoparticle-based cutaneous melanoma treatments have been shown to have increased therapeutic efficacy, improved survival, and reduced tumor recurrence compared with current nonsurgical therapies in selected studies. However, this is limited by the small number of studies, heterogeneous interventions, and limited robust comparative data.

## Introduction and background

The number of new melanoma cases in 2020 was approximately 324,635 worldwide and 105,172 in North America [1]. Although it is the least common skin cancer, it is responsible for the majority (>80%) of skin cancer deaths [2]. ​​Melanoma is a malignancy of melanocytes, which are involved in melanogenesis, the synthesis of melanin pigment within melanosomes [3]. Genetic mutations trigger pathogenic growth of atypical melanocytes through multiple signaling pathways, particularly mitogen-activated protein kinase, MITF, and phosphatidylinositol 3-kinase/PTEN/AKT [4,5]. Early melanoma lesions are confined to the epidermis and have a dark, irregular shape. The tumor progresses to invade the dermis vertically, followed by the subcutis, forming nodules and papules [6,7].​​ Lesions can develop on any part of the skin. Diagnosing melanoma relies on the patient’s history [8]. Examinations are done thoroughly under proper lighting to look for patterns of nevi and discoloration following the Asymmetry, Border irregularity, Color variegation, Diameter >6 mm, and Evolution criteria [9]. Dermoscopy is sensitive and specific for melanoma diagnosis and helps the examiner visualize the lesion without external influences that may affect its appearance [10]. Biopsies, histopathology, and immunohistochemistry tests are also performed to confirm the diagnosis. Current treatment options include chemotherapy, immunotherapy, radiotherapy, phototherapy, and targeted therapies. Melanoma generally has a good prognosis if diagnosed early and surgically removed [11]. However, the prognosis becomes poor if cancer metastasizes [12]. Stage I or II melanoma patients have an approximate five-year survival rate of around 99.4%, which declines to stage 68% in stage III disease and 29.8% in stage IV [13]. Thus, cutaneous melanoma is a public health concern with major psychosocial impacts and requires significant technologies and treatment investments, especially for malignant melanoma.

Nanodrug delivery uses nanoparticles, which are materials measured in the nanoscale. Nanoparticles can be classified into different classes based on their size, shape, chemical properties, and materials, such as metals, polymers, and dendrimers [14]. They function through passive and/or active targeting of the tumor blood vessels. These vessels differ from others in that they contain fenestrated vasculature, allowing nanoparticles to infiltrate the tumor interstitial space. Tumor neovascularization is characterized by enhanced permeability and retention (EPR) effect [15]. The process by which EPR is enhanced, and nanoparticles accumulate in the tumor, is called passive targeting. It is based on the tumor’s leaky vasculature and impaired lymphatic drainage, which helps drug accumulation [15]. Active targeting uses ligands, such as monoclonal antibodies or peptides, to bind to specific receptors on target cells. This mechanism enhances the selectivity of targeting tumor cells through the selective delivery of medications to tumor cells or the tumor microenvironment. This increases the efficacy of the specific treatment of tumor cells while keeping healthy tissue intact. Furthermore, nanoparticles enhance the solubility of poorly water-soluble medications, increase bioavailability, and reduce the number and/or severity of adverse effects associated with cancer drugs. Nanoparticles modify drug distribution and clearance, allowing for the administration of lower doses of other drugs used in cancer treatment [16]. Multiple types of nanomaterials have been studied in clinical trials, ranging from drug delivery, immunotherapy, vaccine development, and imaging. In particular, lipid-based, polymer-based, and inorganic nanocarriers have been investigated for melanoma. However, not many clinical trials have evaluated the therapeutic efficacy of nanoparticles in treating patients with cutaneous melanoma. The types of nanoparticles investigated in this review are polymeric nanoparticles, which can bind or carry the anticancer drug. They have several advantages, including controlled drug release, targeted delivery, and drug solubilization [17]. The next type is virus-like nanoparticles (VLPs) or protein structures resembling wild-type viruses. VLPs have no viral genome nor infectious ability, making them safer candidates for vaccines [18].

This study aims to summarize and discuss the therapeutic efficacy of nanoparticles compared with traditional nonsurgical therapies in reducing tumor recurrence and increasing cancer-specific survival among patients with cutaneous melanoma.

## Review

Methods

Inclusion Criteria and Identification of Studies

The first step in the process was to complete a protocol outlining the systematic review. The review was conducted according to PRISMA guidelines [19]. The National Library of Medicine, through PubMed, Scopus, and the Cochrane Library, searched for melanoma (Medical Subject Headings (MeSH)), nanodrug delivery (MeSH), and nonsurgical treatments (MeSH), combined using Boolean operators. Our inclusion criteria for the studies were clinical trials, randomized controlled trials (RCTs), and non-RCTs. Other criteria for the articles included the English language and publication between 2008 and 2023 (Table 1). Other resources such as Google Scholar, Clinicaltrials.gov, and the Journal of Nanoparticle Research were searched. References from the identified review articles were searched for any additional articles that can be used in the review.

**Table 1:** Search strategy

Database	Search terms	Hits
Pubmed	(((((("nano*"[Title/Abstract] OR "nanoparticles"[Title/Abstract] OR "nanotechnology"[Title/Abstract] OR "nanomedicine"[Title/Abstract] OR "Nano Drug Delivery"[Title/Abstract]) AND "Non-Surgical Treatments"[Title/Abstract]) OR "Skin Cancer Treatments"[Title/Abstract] OR "injections"[Title/Abstract] OR "vaccines"[Title/Abstract] OR "topical treatments"[Title/Abstract] OR "chemotherapy"[Title/Abstract] OR "photodynamic therapy"[Title/Abstract] OR "radiotherapy"[Title/Abstract]) AND "Skin cancer"[Title/Abstract]) OR "melanoma"[Title/Abstract] OR "Malignant melanoma"[Title/Abstract] OR "Cutaneous melanoma"[Title/Abstract] OR "Superficial spreading melanoma"[Title/Abstract] OR "Nodular melanoma"[Title/Abstract] OR "Lentigo maligna melanoma"[Title/Abstract] OR "Acral lentiginous melanoma"[Title/Abstract]) AND (("randomized controlled trial"[Publication Type] OR "controlled clinical trial"[Publication Type] OR "randomized"[Title/Abstract] OR "placebo"[Title/Abstract] OR "drug therapy"[MeSH Subheading] OR "randomly"[Title/Abstract] OR "trial"[Title/Abstract] OR "groups"[Title/Abstract]) NOT ("animals"[MeSH Terms] NOT "humans"[MeSH Terms])) AND ("randomized controlled trial"[Publication Type] AND "english"[Language]) AND ((clinicaltrial[Filter]) AND (2008:2023[pdat]))	825
Cochrane	(Nano* OR nanoparticles OR nanotechnology OR nanomedicine OR nano drug delivery):ti,ab,kw AND (Non-surgical treatments OR skin cancer treatments OR injections OR vaccines OR topical treatments OR chemotherapy OR photodynamic therapy OR radiotherapy):ti,ab,kw AND (Skin cancer OR melanoma OR malignant melanoma OR cutaneous melanoma OR superficial spreading melanoma OR nodular melanoma OR lentigo maligna melanoma OR acral lentiginous melanoma):ti,ab,kw	101
SCOPUS	( TITLE-ABS-KEY ( "clinical trials" OR "clinical trials as a topic" OR "randomized controlled trial" OR "Randomized Controlled Trials as Topic" OR "controlled clinical trial" OR "Controlled Clinical Trials" OR "random allocation" OR "Double-Blind Method" OR "Single-Blind Method" OR "Cross-Over Studies" OR "Placebos" OR "multicenter study" OR "double blind procedure" OR "single blind procedure" OR "crossover procedure" OR "clinical trial" OR "controlled study" OR "randomization" OR "placebo" OR "clinical trials" OR "clinical trials as a topic" OR "randomized controlled trial" OR "Randomized Controlled Trials as Topic" OR "controlled clinical trial" OR "Controlled Clinical Trials as Topic" OR "random allocation" OR "randomly allocated" OR "allocated randomly" OR "Double-Blind Method" OR "Single-Blind Method" OR "Cross-Over Studies" OR "Placebos" OR "cross-over trial" OR "single blind" OR "double blind" OR "factorial design" OR "factorial trial" OR clinical AND trial* OR trial* OR rct* OR random* OR blind* ) AND TITLE-ABS-KEY ( nano* OR nanoparticles OR nanotechnology OR nanomedicine OR "Nano Drug Delivery" ) AND TITLE-ABS-KEY ( "Non-Surgical Treatments" OR "Skin Cancer Treatments" OR injections OR vaccines OR "topical treatments" OR chemotherapy OR "photodynamic therapy" OR radiotherapy ) AND TITLE-ABS-KEY ( "Skin cancer" OR melanoma OR "Malignant melanoma" OR "Cutaneous melanoma" OR "Superficial spreading melanoma" OR "Nodular melanoma" OR "Lentigo maligna melanoma" OR "Acral lentiginous melanoma" ) ) AND ( LIMIT-TO ( DOCTYPE , "ar" ) ) AND ( LIMIT-TO ( SUBJAREA , "MEDI" ) OR LIMIT-TO ( SUBJAREA , "BIOC" ) OR LIMIT-TO ( SUBJAREA , "PHAR" ) OR LIMIT-TO ( SUBJAREA , "IMMU" ) OR LIMIT-TO ( SUBJAREA , "CHEM" ) OR LIMIT-TO ( SUBJAREA , "HEAL" ) ) AND ( LIMIT-TO ( PUBYEAR , 2023 ) OR LIMIT-TO ( PUBYEAR , 2022 ) OR LIMIT-TO ( PUBYEAR , 2021 ) OR LIMIT-TO ( PUBYEAR , 2020 ) OR LIMIT-TO ( PUBYEAR , 2019 ) OR LIMIT-TO ( PUBYEAR , 2018 ) OR LIMIT-TO ( PUBYEAR , 2017 ) OR LIMIT-TO ( PUBYEAR , 2016 ) OR LIMIT-TO ( PUBYEAR , 2015 ) OR LIMIT-TO ( PUBYEAR , 2014 ) OR LIMIT-TO ( PUBYEAR , 2013 ) OR LIMIT-TO ( PUBYEAR , 2012 ) OR LIMIT-TO ( PUBYEAR , 2011 ) OR LIMIT-TO ( PUBYEAR , 2010 ) OR LIMIT-TO ( PUBYEAR , 2009 ) OR LIMIT-TO ( PUBYEAR , 2008 ) ) AND ( LIMIT-TO ( LANGUAGE , "English" ) )	131

The inclusion criteria included different types of cutaneous melanoma patients, including superficial spreading, nodular, lentigo maligna, and acral lentiginous melanoma. The International Classification of Diseases codes C43-C44 were also included to narrow the results to melanoma. Studies that discussed nanoparticles only for diagnosis were excluded: Population: cutaneous melanoma patients (superficial spreading, nodular, lentigo maligna, and acral lentiginous melanoma); Intervention: nanodrug delivery/nanoparticles; Comparison: traditional chemotherapy, radiotherapy, phototherapy, topical medications, injections, and vaccines; Outcome(s): tumor recurrence (primary) and cancer-specific survival (secondary).

Study Selection

The studies were imported into Covidence software (Veritas Health Innovation, Melbourne, Australia) for screening, and duplicates were removed. Titles and abstracts were screened using the inclusion and exclusion criteria, and then full-text screening was conducted. Articles were excluded if they did not have full-text PDFs. Clinical trials were included if they used nonsurgical treatments (chemotherapy, radiotherapy, phototherapy, injections/vaccines, immunotherapy, or topical medications) combined with nanoparticles that included tumor recurrence as a primary outcome and/or cancer-specific survival as a secondary outcome, in patients with cutaneous melanoma.

Data Extraction

Data that met the inclusion criteria were extracted by the coauthors and recorded on a standardized electronic data-collection sheet. The following data were extracted from the included articles: study ID, first author, study type, study aim, participant description, withdrawals and exclusions, type of nanoparticle used, type of cutaneous melanoma treatment used, method of administering the intervention, and types of outcome measures.

Quality Assessment

The quality of clinical data and subsequent recommendations for the treatment of cutaneous melanoma patients were assessed using the Cochrane risk-of-bias tools by two authors independently, with discrepancies resolved after a joint review of the articles and discussion. The Cochrane RoB 1 risk-of-bias assessment tool was used for randomized studies [20], and the Risk of Bias for Included Non-Randomized Studies-I tool was used for nonrandomized studies [21]. The results of each assessment tool are displayed in Tables 2, 3, respectively. In addition, the Oxford Center for Evidence-Based Medicine: Levels of Evidence was used to assess the strength of the included papers. A PRISMA checklist was done for completeness. The studies included were Speiser et al. [22], Goldinger et al. [23], Kottschade et al. [24,25], Alrwas et al. [26], and Hersh et al. [27,28].

**Table 2:** Risk of Bias for included randomized studies (risk of bias 1)

Parameter	Speiser et al. [22]	Kottschade et al. [25]	Hersh et al. [28]
Random sequence generation (selection bias)	Low	Low	Low
Allocation concealment (selection bias)	High	Low	Low
Blinding of participants and personnel (performance bias)	High	Low	High
Blinding of outcome assessment (detection bias)	High	Low	High
Incomplete outcome data (attrition bias)	Low	Low	Low
Selective reporting (reporting bias)	Low	Low	Low
Other bias (evidence selection bias/confirmation bias)	Unclear	Unclear	Unclear
Overall risk of bias	High	Low	High

**Table 3:** Risk of bias for included nonrandomized studies

Parameter	Goldinger et al. [23]	Kottschade et al. [24]	Alrwas et al. [26]	Hersh et al. [27]
Baseline confounding	Serious	Serious	Serious	Moderate
Selection of participants	Moderate	Moderate	Moderate	Moderate
Classification of interventions	Low	Low	Low	Low
Deviation from intended interventions	Low	Low	Low	Moderate
Missing data	Moderate	Moderate	Low	Moderate
Measurement of outcomes	Moderate	Low	Moderate	Moderate
Selection of reported results	Low	Moderate	Low	Moderate
Overall risk of bias	Serious	Serious	Serious	Moderate

Results

**Figure 1:**
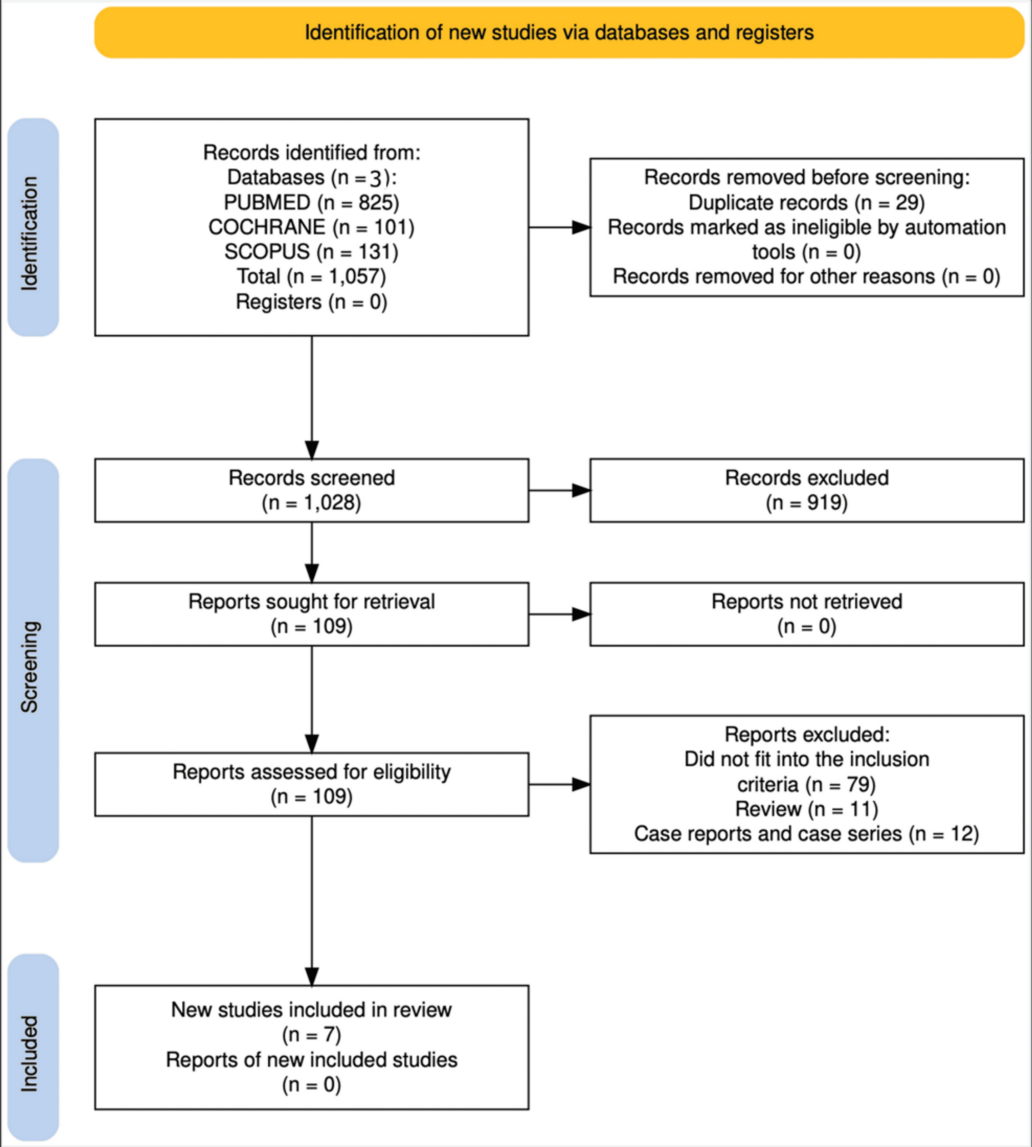
PRISMA flow diagram for study selection

After applying our search strategy, 1,057 studies were screened and assessed for eligibility. Seven studies (four non-RCTs and three RCTs) with 825 adult patients were included (Figure 1). The main category of patients had stage III or IV melanoma. There were a variety of outcome measures, including T-cell immune response, progression-free survival (PFS), overall survival (OS), tumor response rate, and toxicity. Multiple chemotherapy and immunotherapy drugs for melanoma, combined with nanoparticles, were studied (Table 4). The studies included show heterogeneity regarding efficacy and treatment outcomes. However, they were brought together under a single review question to highlight recent advances in the field of melanoma treatment.

**Table 4:** Charted data for each study highlighting nanoparticle treatment, primary outcomes, and key findings RCT: randomized controlled trial; VLP: virus-like nanoparticle; IFA: incomplete Freund’s adjuvant; ISA-51: incomplete seppic adjuvant 51; RECIST: response evaluation criteria in solid tumors; PFS: progression-free survival; OS: overall survival; PT: previously treated; CN: chemotherapy naive; TB: temozolomide/bevacizumab; AUC: area under the curve; CNS: central nervous system; ORR: overall response rate; DCR: disease control rate

Study	Study design	Study sample	Study population	Nanoparticle treatment	Primary outcome	Evidence level	Key findings
Speiser et al. [22]	3 individual substudies (2 single-group and 1 RCT)	22	Stage II-IV melanoma patients	Virus-like nanoparticle. MelQbG10 vaccination (subcutaneous or intradermal)	Cellular immune response and safety. An exploratory design was used to test clinical efficacy	2B	In this study, it was found that VLPs drain efficiently into the lymph nodes, proving the efficiency of QbG10 as a carrier of the vaccine. VLP was combined with Melan-A_16-35_ A27L analog peptide to form the vaccine MelQbg10. Vaccination resulted in detectable CD8 T cells in most patients in all stages of metastatic melanoma
Goldinger et al. [23]	Non-RCT	21	Stage III-IV melanoma patients were recruited	Virus-like nanoparticle. MelQbG10 vaccine WITH IFA (Montanide) s.c. (Group I). MelQbG10 vaccine WITH IFA (Montanide) s.c. AND topical imiquimod (Group II). MelQbG10 vaccine given i.d. WITH topical imiquimod (Group III). MelQbG10 vaccine as an intra-lymph node injection (Group IV)	To increase the MelQbg10 vaccine’s immunogenicity by using Montanide ISA-51 (IFA), imiquimod 5% topical cream that activates antigen-presenting cells	2B	17/21 patients completed the study according to protocol, 4/21 discontinued due to disease progression. Humoral responses were developed in all patients. All patients developed Melan-A and Qb-specific IgG antibodies. Groups I and II showed much higher titers than groups III and IV. The disease progression evaluation (first follow-up) could only be completed for 14/21 (66%) patients. 9/14 (64%) documented to have progressive disease, and 5/14 (36%) documented to have stable disease
Kottschade et al. [24]	Non-RCT	76	Unresectable stage IV malignant melanoma	Polymeric nanoparticle. carboplatin (IV) AND paclitaxel albumin-stabilized nanoparticle formulation (ABI-007) (IV)	Objective response rate (RECIST), PFS, and OS	1B	34/73 patients were in PT (cohort 1), 39/73 patients in CN (cohort 2). Three responses (8.8%) in the PT cohort (90% CI: 2.5-21.3), median PFS was 4.1 months, and median OS was 10.9 months. Cohort 2: 10 responses (25.6%) in CN cohort (90% CI: 16.7-42.3), median PFS was 4.5 months, and median OS was 11.1 months. Treatment was discontinued in 28/39 (72%) patients due to the progression of the disease
Kottschade et al. [25]	RCT	93	CN patients with unresectable stage IV malignant melanoma	Polymeric nanoparticle. Regimen TB: temozolomide (200 mg/m^2^ orally days 1-5) AND bevacizumab (10/kg IV days 1 and 15 of a 28-day cycle). Regimen ABC: Nab-paclitaxel (100 mg/m^2^ IV days 1, 8, and 15) AND bevacizumab (10 mg/kg IV days 1 and 15) AND carboplatin (at AUC of 6 IV on day 1, of a 28-day cycle)	PFS rate, and OS	1B	Regimen TB-42 patients. 32/42 discontinued treatment due to disease progression. 3/42 patients discontinued treatment due to grade 3 adverse events. Median PFS time and median OS with TB were 3.8 and 12.3 months, respectively. Regimen ABC-51 patients. 12/51 patients discontinued treatment due to grade 3 adverse events. 28/51 discontinued treatment due to disease progression. The median PFS and OS times with ABC were 6.7 and 13.9 months, respectively. The addition of bevacizumab to nab-paclitaxel and carboplatin shows promising activity despite tolerability issues
Alrwas et al. [26]	Non-RCT	10	Stage IV or unresectable stage III metastatic melanoma patients	Polymeric nanoparticle. Biochemotherapy: (IV cisplatin (20 mg/m^2^), subcutaneous interferon-α (5 × 106 IU/m^2^), continuous IV interleukin-2 (9 × 106 IU/m^2^)] WITH oral temozolomide (250 mg/m^2^) AND Nab-paclitaxel	Safety and toxicity of biochemotherapy with temozolomide and nab-paclitaxel, and maximum tolerated dose of nab-paclitaxel	2B	Secondary aim: response rate and duration, OS, and incidence of CNS metastasis. The median time to disease progression was 5.3 months, and the median OS was 8.73 months. 5/9 (56%) of patients had a partial response. 0 patients showed a complete response to treatment. 6/9 patients developed melanoma metastasis in the CNS. The results overall were that biochemotherapy with nab-paclitaxel and temozolomide resulted in significant toxicity in patients with metastatic melanoma
Hersh et al. [27]	Non-RCT	74	Confirmed malignant melanoma patients overall were enrolled. Cohort 1: previously received chemotherapy. Cohort 2: CN patients	Polymeric Nanoparticle. Cohort 1- Nab-paclitaxel (ABI-007) IV weekly for 3 weeks at 100 mg/m^2^. Cohort 2- Nab-paclitaxel (ABI-007) IV weekly for 3 weeks at 150 mg/m^2^	ORR, PFS, OS	2B	Cohort 1%-5% of patients have experienced grade 3 or 4 sensory neuropathy. ORR was 2.7%, response plus stable disease rate was 37.8%, median PFS was 3.5 months, and median OS was 12.1 months. Cohort 2: 19% of patients have experienced grade 3 or 4 sensory neuropathy. ORR was 21.6%, significantly higher than that of the patients in the first cohort. Response plus stable disease rate was 48.6%, and median PFS was 4.5 months. Nab-paclitaxel is well tolerated and has shown favorable results compared to the current gold standard, dacarbazine therapy, as well as other combination therapies
Hersh et al. [28]	RCT	529	CN Stage IV patients were recruited and randomized to receive either nab-paclitaxel (264) or dacarbazine (265 patients)	Polymeric nanoparticle. Nab-paclitaxel (150 mg/m^2^ on days 1, 8, and 15 every 4 weeks). Dacarbazine (1,000 mg/m^2^ every 3 weeks)	PFS, OS, ORR, DCR	1 1B study, RCT	Progression-free survival (PFS): 4.8 months with nab-paclitaxel vs. 2.5 months with dacarbazine. OS: 12.6 months nab-paclitaxel vs. 10.5 months dacarbazine. ORR: 15% nab-paclitaxel vs. 11% dacarbazine (p = 0.239). DCR: 39% nab-paclitaxel vs. 27% dacarbazine (p = 0.004). Neuropathy (nab-paclitaxel, 25% vs. dacarbazine, 0%; p < 0.001) and neutropenia (nab-paclitaxel, 20% vs. dacarbazine, 10%; p = 0.004) were the common grade ≥3 treatment-related adverse events

VLP for Melanoma Treatment

Speiser et al. used a VLP combined with the Melan-A16-35 A27L analog peptide to form the vaccine MelQbg10, which induced ex vivo-detectable CD8 T-cell responses in most stage II-IV melanoma patients. They concluded that vaccination with CpG-loaded VLPs can successfully induce human CD8 T-cell responses and potential long-term immunity from the disease, an essential requirement for immunotherapy [22].

To further increase the immunogenicity of the MelQbg10 vaccine, Goldinger et al. investigated the MelQbG10 vaccine with incomplete Freund’s adjuvant (IFA; Montanide), the MelQbG10 vaccine with IFA and topical Imiquimod, the MelQbG10 vaccine with topical 5% Imiquimod cream alone, and administering the vaccine as an intra-lymph node injection. There was a 76% response rate to the MelQbG10 vaccine with a twofold increase in the percentage of Melan-A tetramer-positive cells. T-cell frequencies were significantly increased in Group I and II patients after administration of MelQbg10 with IFA (Montanide) compared with patients receiving vaccines without IFA. The combination of MelQbg10 and Imiquimod with IFA triggers toll-like receptor (TLR)-7 and TLR-9, leading to enhanced promotion of memory and effector-phenotype cells. This is significant since many relapsing melanoma cases show decreased expression of Melan-A or human leukocyte antigen class I, demonstrating an immune escape mechanism. This highlights the importance of immunotherapy as a treatment for relapsing melanoma. Overall, the study demonstrated that MelQbG10 is safe and well-tolerated at a cumulative dose of 6 mg. Although the combination of IFA with MelQbG10 caused more frequent side effects, it also induced the highest Melan-A-specific T-cell frequencies. Future vaccines need to be able to target multiple antigens and be tested on humans to determine which formulations can provide the most clinical benefit for patients [23]. Both studies highlight that VLP-based vaccines are able to generate T-cell responses, providing protection from malignant diseases, meaning they can be used in clinical practice.

Human Albumin Nanoparticle for Melanoma Treatment

Nab-paclitaxel and carboplatin: Nab-paclitaxel (ABI-007) is a solvent-free, albumin-bound nanoparticle formulation of the chemotherapy drug paclitaxel created to reduce the Cremophor vehicle-associated toxicities of paclitaxel. To examine nab-paclitaxel’s antitumor properties, Kottschade et al. combined nab-paclitaxel with carboplatin. They studied the objective response rate (response evaluation criteria in solid tumors) in patients with unresectable stage IV melanoma who were either chemotherapy-naive (CN) or previously treated (PT). The advantages of using ABI-007 instead of paclitaxel combined with carboplatin are the ability of ABI-007 to deliver a higher dose of paclitaxel and show higher response rates, a shorter time to disease progression, and a lower incidence of neutropenia. ABI-007 can also bind secreted protein acidic and rich in cysteine, a protein on the surface of malignant melanocytes, enhancing ABI-007 delivery to the tumor site at higher concentrations. They discovered that weekly administration of ABI-007, combined with carboplatin, seems to be well tolerated with promising therapeutic activity in CN patients, and modest antitumor activity in PT patients. For CN patients who are unable to tolerate paclitaxel therapy, the combination of ABI-007 and carboplatin appears effective, although such formulations should be compared in future RCTs [24].

Nab-paclitaxel, carboplatin, temozolomide, and bevacizumab: In another study, Kottschade et al. investigated combining chemotherapy (nab-paclitaxel with carboplatin or temozolomide) and immunotherapy (bevacizumab) for CN patients with unresectable stage IV malignant melanoma. They found higher efficacy and a median OS of 13.9 months in the bevacizumab-nab-paclitaxel with carboplatin treatment cohort, vs. 12.3 months in the temozolomide and bevacizumab group [25].

Nab-paclitaxel, temozolomide, and biochemotherapy: Alrwas et al. used nab-paclitaxel and temozolomide with biochemotherapy (cisplatin, interferon-α, and interleukin-2) for metastatic melanoma to study its safety, toxicity, and maximum tolerated dose (MTD) of nab-paclitaxel. They also investigated substituting nab-paclitaxel with less potent substances to look for an increased response rate and duration. They were unsuccessful in finding the MTD of nab-paclitaxel due to dose-limiting toxicities. Although the combination elicited responses outside the central nervous system, they noted that biochemotherapy with nab-paclitaxel and temozolomide caused significant toxicity [26]. However, this study is limited by the lack of MTD for nab-paclitaxel and the small number of patients enrolled, which may affect the accuracy of its efficacy and survival evaluations. Therefore, future clinical trials need to be conducted to determine the MTD of nab-paclitaxel combined with biochemotherapy to recommend an effective treatment regimen for metastatic melanoma patients.

Nab-paclitaxel in CN and treated patients: Hersh et al. studied two cohorts: ABI-007 in inoperable locally recurrent or metastatic melanoma patients who had previously received chemotherapy and in CN patients for the response rate, PFS, and OS. They found that nab-paclitaxel was well tolerated and showed activity in both cohorts. It showed favorable results compared with the current gold standard dacarbazine therapy, previous studies of single-agent paclitaxel therapy, combination chemotherapy, and other therapies for PT melanoma patients. The median OS in the PT vs. CN patients was 12.1 and 9.6 months, respectively. This is significantly longer than the median survival of less than eight months reported for patients treated with dacarbazine or temozolomide. The PFS in chemotherapy-PT vs. CN patients was 3.5 and 4.5 months, respectively, which is longer than the PFS of 1.6 months in patients treated with dacarbazine or temozolomide. Considering metastatic melanoma has a short survival and an unrelenting disease progression, this study shows the effectiveness of using nab-paclitaxel to slow disease progression and extend patient survival time [27].

In a different study by Hersh et al., patients with randomized CN metastatic melanoma were treated with nab-paclitaxel or dacarbazine to assess the PFS, OS, and disease control rate (DCR). They found that nab-paclitaxel significantly improved PFS and DCR compared with dacarbazine, with a manageable safety profile [28]. The higher efficacy of nab-paclitaxel compared with solvent-based (sb) paclitaxel can be attributed to the unique pharmacokinetic profile of albumin-based nab technology and the lack of solvent, which alone causes neuropathy and hypersensitivity reactions. Thus, nab technology can contribute to an enhanced tolerability profile and to higher doses and intensities of nab-paclitaxel compared with sb-paclitaxel [29-35]. Both studies highlight the importance of nab-paclitaxel as a treatment option for BRAF wild-type and mutant melanoma, as well as CN patients with metastatic melanoma.

Discussion

This review found that the MelQbg10 vaccine elicited positive treatment responses, fewer adverse effects, and increased survival rates. The nab-paclitaxel chemotherapy drug combinations were effective but associated with toxicities. The evidence suggests that nanoparticles, in combination with melanoma drugs, have the potential to be used for treatment. However, due to the limited RCTs conducted, a definitive conclusion cannot be made.

Limitations, Strengths, and Weaknesses

The main strength of this article is the methodology. Details were provided regarding the search strategy, study selection, data extraction, and databases searched. The risk of bias was also assessed and clearly displayed for each study. The included studies enrolled a limited number of patients to test the therapeutic efficacy of nanoparticles for cutaneous melanoma treatment. There were even fewer RCTs. Conducting more RCTs is therefore necessary to determine the safety, toxicity, and efficacy profiles of nanoparticle drug formulations. Furthermore, there was a high risk of performance, detection, and selection biases regarding blinding and randomization across the studies. With the nonrandomized studies, there was a high risk of bias regarding baseline confounding and participant selection. The high risk of bias among the studies poses a significant limitation to our study, as it affects the confidence in the safety and efficacy of these interventions. In addition, this systematic review examined only two main types of nanoparticles used in cutaneous melanoma treatments: polymersomes and VLPs, as these were used in clinical trials. Future research needs to be conducted in a clinical trial setting with different combinations of nanoparticles and cutaneous melanoma drugs to find the best combination.

Implications

According to the European Society for Medical Oncology Clinical Practice Guidelines, nanoparticles are not currently listed as a standard treatment. The first-line treatment for unresectable stage III and IV melanoma includes anti-PD-1 drugs, anti-PD-1 drugs with anti-cytotoxic T-lymphocyte-associated Protein 4 drugs (e.g., ipilimumab), a BRAF inhibitor (e.g., vemurafenib) combination, and MEK inhibitors for BRAF-mutated melanoma [36]. Kottschade et al. found that the combination of bevacizumab with nab-paclitaxel and carboplatin was effective in increasing PFS and OS, comparable to that of drugs vemurafenib and ipilimumab for metastatic melanoma. Thus, this treatment combination should be explored in further phase III clinical trials to implement it in clinical practice guidelines for metastatic melanoma. The National Comprehensive Cancer Network guidelines recommend nab-paclitaxel alone for the treatment of metastatic or advanced melanoma. To expand on this recommendation, clinical trials of nab-paclitaxel combined with targeted immunotherapies are needed. While the use of nanoparticles in practice is relatively new, it is a field needing further research and development to determine its role in modern melanoma treatment.

Finally, our findings suggest that nanoparticles can be used in parallel with the treatments included in the guidelines to enhance their efficacy [37]. VLPs combined with Melan-A16-35 A27L analog peptide can be administered as vaccines to stimulate an immune response. Nab-paclitaxel can be used in conjunction with chemotherapy drugs such as carboplatin, as well as immunotherapies such as bevacizumab. Nanoparticles are advantageous in the sense that they can overcome certain barriers that traditional melanoma therapies cannot. Systemic chemotherapy causes many side effects due to its lack of targeting and widespread distribution around the body. There is also the issue of tumor resistance to chemotherapy drugs [38]. Nanoparticles can transport the drug directly to the tumor through both passive and active targeting. Targeted therapy and immunotherapy are costly and associated with low patient compliance [39]. Topical treatments have limited permeability of the stratum corneum, limiting the amount of drug reaching the tumor. Nanoparticles have features that can overcome these barriers, including improved skin permeability and drug retention [40], as well as evading the reticuloendothelial system [41].

## Conclusions

Nanoparticles are promising vehicles for developing future melanoma treatments, particularly for metastatic melanoma. This review found that the MelQbg10 vaccine and combinations of nab-paclitaxel elicited positive treatment responses, fewer adverse side effects, and increased PFS and OS rates. However, this review is limited by a high risk of bias, a small number of studies, and heterogeneity in interventions and outcomes. Therefore, further clinical and comparative trials are needed to specify ideal nanoparticle combination therapies for cutaneous melanoma patients.
